# Inhibition of the Prostaglandin EP2 Receptor Prevents Cognitive Impairment in a Model of Bacterial Sepsis

**DOI:** 10.21203/rs.3.rs-11125792/v1

**Published:** 2026-10-30

**Authors:** Chunxiang Jiang, Radhika Amaradhi, Thota Ganesh, Dingledine Ray

**Affiliations:** Emory University; Emory University; Emory University; Emory University

**Keywords:** Sepsis associated encephalopathy, EP2 antagonist, Novel object recognition, Cognitive impairment, Inflammation

## Abstract

Sepsis is a life-threatening syndrome caused by a dysregulated host response to infection and can produce sepsis-associated encephalopathy (SAE), including acute delirium and longer-term cognitive impairment. Prostaglandin-E2 signaling through the EP2 receptor contributes to neuroinflammation and cognitive dysfunction in several experimental settings, but its role in post-sepsis cognitive impairment is unclear. Here, we characterize the inflammatory profile of hippocampus, kidney and blood after cecum ligation and puncture (CLP), which induces sepsis. An EP2 receptor antagonist was administered after CLP, and recognition memory was assessed seven days later by the novel object recognition test. Levels of the synaptic proteins, PSD-95 and synaptophysin, were also evaluated by western blot. CLP produced transient hypothermia, progressive weight loss, sickness behavior, splenomegaly, altered blood-cell counts, and persistent elevations in neutrophil-to-lymphocyte and monocyte-to-lymphocyte ratios. TNFα and CCL2 mRNA levels were persistently elevated in hippocampus and there was a larger and broader cytokine induction in kidney. Administration of EP2 antagonist TG6–10-1 at 2 and 26 h after CLP did not significantly alter survival, body weight, sickness behavior, or peripheral inflammatory measures. Nevertheless, TG6–10-1 prevented the CLP-associated deficit in novel object recognition and prevented the reduction in cortical PSD-95 abundance observed at day 7. These findings identify EP2 receptor signaling as a contributor to cognitive impairment after CLP and support further investigation of EP2 antagonism as a potential strategy for SAE-related cognitive dysfunction.

## Introduction

Sepsis is a multi-organ dysfunction caused by a dysregulated response to infection (Singer et al., 2016) and is a major cause of ICU admission and death worldwide. The brain has been reported to be among the first organs affected during sepsis (Mazeraud et al., 2016) and the incidence of sepsis associated encephalopathy (SAE, a neurologic complication with in sepsis) is approximately 50% (Li et al., 2025;Mazeraud et al., 2020). In addition to delirium and coma in the acute phase of SAE, patients with sepsis are at increased risk of long-term cognitive impairment (Basu et al., 2025;Iwashyna et al., 2010;Payal et al., 2025), depression (Mazeraud, et al., 2020), anxiety (Nikayin et al., 2016) and seizures (Reznik et al., 2017). Up to 51% of septic patients fail to return to work or study after one year (Herridge et al., 2003). Enhancing the recovery of brain function can help sepsis survivors live independently and reduce the social healthcare burden. However, post-sepsis recovery therapy is limited to rehabilitation (Bircak-Kuchtova et al., 2025), and there is no evidence-based pharmacotherapy that can improve the long-term cognitive outcome of SAE patients.

Prostaglandin-E_2_ (PGE_2_), a significant product of cyclooxygenase-2 (COX-2) (Liang et al., 2007;Rojas et al., 2019), plays a pivotal role in regulating the inflammatory response. There are four downstream G-protein coupled receptors (GPCRs) for PGE_2_: EP1, EP2, EP3 and EP4 (Sugimoto and Narumiya, 2007). Previous studies have reported that EP2 antagonist treatment could reduce neuroinflammation (Jiang J. et al., 2019) and neurodegeneration, protect blood brain barrier (Rojas et al., 2020) and prevent memory impairment (Rojas et al., 2016;Varvel et al., 2023) in different animal models of prolonged seizure.

Cecum ligation and puncture (CLP) is a frequently used rodent model to study sepsis and explore new therapies. CLP is a polymicrobial infection model that recapitulates several key features of clinical sepsis. A selective EP2 antagonist was shown to reduce intestinal COX-2 expression after CLP (Golden et al., 2020), indicating that PGE_2_ may exert its function partly via the EP2 receptor in the CLP mouse model. Moreover, global knockout of EP2 receptors increased the survival rate of septic mice induced by intrauterine Streptococcus pyogenes infection (Mason et al., 2013), which suggests that inhibition of EP2 receptors may be beneficial to sepsis.

Surviving rodents after CLP showed memory deficits in the novel object recognition (NOR) test (Akhondzadeh et al., 2020), water-maze test (Zhang et al., 2020), and Y-maze test (Liu et al., 2014). Among them, NOR is relatively low stress and is sensitive to dysfunction involving cortical and hippocampal circuits (Bellantuono et al., 2020). We previously showed that pharmacological blockade of EP2 receptors could reduce cortex COX-2 protein expression, reverse the reduction of synaptic proteins in cortex, and prevent memory deficit in a mouse model of LPS-induced peripheral inflammation (Jiang Chunxiang et al., 2020). It is still unclear whether an EP2 antagonist can improve the cognitive deficit of mice subjected to CLP, a more comprehensive model of sepsis than LPS administration. Therefore, a mouse CLP model of sepsis was used to study the effect of a selective, brain permeant EP2 receptor antagonist on memory deficits. We characterized and compared complete blood counts and inflammatory-gene expression in the hippocampus and kidney to assess the temporal and tissue-specific breadth of the inflammatory response. Our results indicate that EP2 receptor inhibition after sepsis initiation prevented the reduction in PSD-95 abundance and eliminated the recognition memory deficit. These results reinforce the idea that EP2 receptors contribute to the development of long-term memory deficit following sepsis.

## Materials and Methods

### Animals

Seven- to ten-week-old adult male C57BL/6N mice (Charles River, United States) were used for experiments. Mice were housed under a 12 hr light-dark cycle with food and water ad libitum. All animal experimental procedures were approved by the Institutional Animal Care and Use Committee (IACUC) of Emory University and conducted in accordance with its guidelines.

### Sepsis model

The CLP procedure was performed as published by Fay et al (Fay et al., 2019). Mice were anesthetized with isoflurane, and a 1 cm midline incision was made in the lower abdomen. The cecum was exposed and ligated 1 cm from its base. The cecum was then punctured through by a 22-gauge needle, and approximately 1mm stool was gently extruded into the abdominal cavity. The ligated cecum was placed back in the abdominal cavity, the midline incision was then sutured closed, and animal surgical adhesive was used to close the skin. A sham group underwent the same procedure without cecum ligation and puncture. All animals received buprenorphine (0.1 mg/kg, sc; Patterson veterinary, USA) preoperatively for pain relief. Fluid resuscitation with 1 ml saline as well as antibiotic therapy (ceftriaxone 50 mg/kg; Sigma, USA and metronidazole 35 mg/kg; Sigma, USA) were administered subcutaneously to mimic the clinical care of patients. Antibiotics were given every 12 hours for 48 hours postoperatively. Animals were monitored every 15 min following anesthesia until they were able to walk. A heating pad was placed under the recovery cage for two days to reduce hypothermia, and animals were checked twice daily.

### EP2 antagonist treatment

Following CLP or sham surgery. mice were randomized to treatment with vehicle (2% dimethylacetamide and 98% corn oil, sc) or EP2 antagonist, TG6–10-1 (10 mg/kg in vehicle, sc). This dose and dose schedule were selected because they maintained adequate brain exposure (concentrations above the Schild Kb) to the EP2 antagonist for > 48 hr (Jiang Chunxiang, et al., 2020). TG6–10-1 or vehicle were administered 2 hr and 26 hr after surgery by an investigator blinded to which solution was used. The blind was maintained throughout behavioral scoring. The sham-vehicle and sham-TG6–10-1 treated mice were pooled before analysis, since previous studies showed no effect of TG6–10-1 alone.

### Open field test and Novel object recognition test

A novel object recognition (NOR) test was used to assess recognition memory in mice that survived as previously reported (Jiang Chunxiang, et al., 2020). There were three stages, including habituation (5 min), training (10 min) and testing (5 min). To reduce stress and anxiety, mice were handled by the same investigator a few minutes each day for seven days before the experiment. Mice were acclimated to the behavior room one hour before testing. The NOR apparatus was 40 cm × 40 cm × 30 cm. ANY-maze, a behavior tracking software, was used to track mice, compute time spent with objects or in the arena center, and distance travelled. On day 6 after surgery, the first habituation was performed as an open field test. Mice were allowed 5 min to explore the arena freely in the absence of objects. On the next day, mice were allowed to explore two identical objects in the arena for 10 min, then removed to their home cage. After 2 hours, one object was replaced by a similar sized novel object and mice were given 5 min to explore freely. Between each test, 70% ethanol solution was used to clean the arena and objects. Before experiments, predefined areas around both novel and known objects were fixed in the arena. The mouse head was tracked. Exploration was defined as the mouse’s head being within 2 cm of an object while sniffing, touching or gnawing it. Excluded from the total exploration time was any time spent with the object where a mouse simply propped the forepaws onto the object with the head pointing away from the object. A trained observer reviewed video recordings to identify behaviors that could not be classified reliably by the tracking software. Mice were excluded if total object-exploration time was less than 15 s, or if they spent more than 75% time on one object in the training stage or only explored one object in the testing stage. With these criteria, 8 of 45 mice were excluded (1 mouse treated with vehicle and 7 mice treated with drug). The higher exclusion rate in TG6–10-1-treated mice was not observed in prior studies in mice or rats using this compound (Jiang Chunxiang, et al., 2020;Rojas, et al., 2016) or two other EP2 antagonists tested in mice (Ganesh et al., 2026;Varvel, et al., 2023); nevertheless, this imbalance should be considered when interpreting the NOR data. A discrimination index was used to evaluate the ability to recognize novel objects.


$$Discrimination\;index=\frac{Time_{novel}\;-\;Time_{familiar}}{Time_{novel}\;+\;Time_{familiar}}$$

### Complete blood count (CBC)

Mice were given an overdose of isoflurane and then terminal blood was collected by cardiac puncture into tubes containing K_2_EDTA. Whole blood was analyzed with a HM5 Hematology Analyzer for blood cell counts.

### RT-qPCR

Mice were perfused with cold PBS and decapitated to obtain the brain. Total RNA of mouse or hippocampus was isolated using TRIzol (Invitrogen) with the PureLink RNA Mini Kit (Invitrogen). RNA concentration and purity were measured by A260 value and A260/A280 ratio, respectively. For cDNA synthesis we used 1 μg of total RNA, 4 μl qScript cDNA Supermix (Bio-Rad Laboratories) in a reaction volume of 20 μl at 25°C for 5 min, 42°C for 30 min, and 85°C for 5 min. Quantitative real-time PCR (qRTPCR) was performed on mixtures of 2 μl 5× diluted cDNA, 1 μl 10 μM each forward and reverse primers, and 10 μl iQ SYBR Green Supermix (Bio-Rad Laboratories) with a final volume of 20 μl in the iQ5 Multicolor Real-Time PCR Detection System (Bio-Rad Laboratories). PCR cycling conditions were as follows: 95°C for 2 min followed by 40 cycles of 95°C for 15 s and 60°C for 1 min. Melting-curve analysis was used to verify single-species PCR product. Fluorescent data were acquired at the 60°C step. The geometric mean of cycle thresholds for GAPDH, β-actin and HPRT1 was used as an internal control for each sample. Primers used for qRT-PCR are in Table 1.

### Western blot

Western blot was done as previously reported (Jiang Chunxiang, et al., 2020). Cortex was homogenized on ice in 0.5 ml RIPA buffer (ThermoFisher Scientific) containing a mixture of protease and phosphatase inhibitors (ThermoFisher Scientific). Protein concentration was measured in the collected supernatant by Bradford assay kit (Bio-rad) after centrifugation (12,000 × g, 15 min, 4°C). 30 μg of total proteins was separated by 4–15% SDS-PAGE and electroblotted onto PVDF membranes (Millipore). The membrane was blocked with 5% non-fat milk at room temperature for one hour, then incubated overnight at 4°C with primary antibodies: rabbit anti-PSD-95 (1:2000, Cell Signaling Technology, 3450), rabbit anti-Synaptophysin (1:2000, Cell Signaling Technology, 5461) and mouse anti-β-actin (1:5000, Abcam, ab6276). After incubating with anti-mouse IRDye 680RD secondary antibody (1:10000) or anti-rabbit IRDye 800CW secondary antibody (1:10000) at room temperature for 1 hour, the blots were washed with 1X Tris-Buffered Saline, 0.1% Tween 20 and scanned. The band intensities were quantified by Image lab (Bio-Rad).

## Statistical analysis

All data are presented as mean ± SEM, or as dot plots, or as box-and-whisker plots (25th–75th quartiles with median as the central line and whiskers spanning the minimum to maximum observed values). All comparisons were pre-specified per domain outcome. Statistical analyses were performed with GraphPad Prism software (version 8.0). One-way or two-way ANOVA with post hoc Bonferroni or Dunnett’s test, or paired or unpaired t-tests were performed as appropriate. Exact p-values are reported; p < 0.05 was considered statistically significant.

## Results

### Bacterial sepsis has myriad effects on the body

Recent studies pointed out that peripheral immunocytes, including monocytes and neutrophils, could migrate into the brain after sepsis and cause brain dysfunction via activating microglia or releasing inflammatory cytokines (Andonegui et al., 2018;He et al., 2016). CBC provides a clinically familiar description of hematologic responses after CLP. It reveals how immunocytes, erythrocytes and platelets respond to sepsis. Thus, terminal blood collection was performed at denoted time points after sham or CLP procedure to study the blood cells. Leukocytes (WBC), neutrophils and monocytes all peaked six hours after CLP, dipped at two days then recovered by seven days after CLP (Fig. 1A-C). Lymphocytes first decreased and then returned to a basal level at day 3 (Fig. 1D). Platelets stayed at a significantly low level between day 1 and day 3 (Fig. 1E) and erythrocytes decreased significantly at day 7 (Fig. 1F). Neutrophil-to-lymphocyte ratio (NLR) is a useful biomarker of severity in patients with sepsis(Ye et al., 2020). Patients who have a higher NLR usually have a poor prognosis. Notably, NLR remained elevated in CLP mice at day 7 (Fig. 1G). Studies on relationship between monocyte-to-lymphocyte ratio (MLR) and sepsis are scarce. However, MLR is also significantly higher in the CLP mice than in the sham mice at day 7 (Fig. 1H).

During sepsis mice became hypothermic, lost weight, developed sickness behaviors and an enlarged spleen (Fig. 2). EP2 antagonist treatment did not influence body weight, survival of mice subjected to CLP and sickness behaviors as evaluated with an Irwin score (Fig. 2A-C). Survival rate is 66.7% in the vehicle treated septic mice, and 80% in the TG6–10-1 treated group. The body temperature of mice at 6h post CLP was significantly lower 0h, which was not impacted by the TG6–10-1 treatment (Fig. 2D). To further characterize the systemic response to CLP, splenic size and weight were measured on day 7. Mice in sham-vehicle group and sham-TG6–10-1 group were considered as one group. As expected, mice in CLP and vehicle group had a heavier and larger spleen than mice in sham group. TG6–10-1 did not alter CLP-associated splenomegaly (Fig. 2E-F), indicating that EP2 antagonism did not measurably alter this index of systemic illness.

### Hippocampus and kidney inflammatory gene expression of septic mice

Neuroinflammation is expected to have an important role in the development of SAE. Since the CLP model shows a milder neuroinflammatory profile compared with the LPS model (Jiang Chunxiang, et al., 2020), gauging the breadth of brain inflammatory response helps us decide how to apply drugs. Meanwhile, it remains unclear whether central and peripheral inflammatory responses occur with similar timing and magnitude. Both hippocampi and kidneys of mice were collected to study the inflammatory gene expression between 6 hour to 168 hour following sepsis. As Fig. 3 shows, mRNA level of hippocampus TNF-α elevated rapidly following sepsis and stayed at a significantly high level at day 7 (Fig. 3A1), whereas kidney TNF-α mRNA peaked at 12 hr and returned to a basal level at day 7 (Fig. 3B1). CCL2 induction was smaller in the hippocampus than in the kidney (Fig. 3A2, B2), although expression remained elevated in both tissues at later time points. Both IL-6 and IL-1β elevated faster and higher in kidney than in hippocampus after CLP, indicating a sensitive response in the peripheral system.

### EP2 antagonist prevents memory deficit following sepsis

Total distance traveled in the open-field session was measured as an index of locomotor activity. Because CLP reduced locomotor activity (Fig. 4A), we used the discrimination index to normalize novel-object preference to total object-exploration time (Bellantuono, et al., 2020); residual non-mnemonic confounding cannot be excluded. The EP2 antagonist did not improve motor ability of survivors following CLP (Fig. 4A). Our previous data showed that the EP2 antagonist prevented memory deficits in the novel object recognition test in both status epilepticus model and LPS model (Jiang Chunxiang, et al., 2020;Rojas, et al., 2016), but it was unclear if an EP2 antagonist can prevent memory deficits in the CLP model. Thus, surviving mice were tested with a novel object recognition test 7 days after sepsis to reveal the effect of EP2 antagonist on cognitive function. As Fig. 4B shows, mice in both sham groups were able to discriminate between the novel and familiar objects, whereas mice in CLP and vehicle treated group did not show a preference for the novel object, consistent with impaired recognition memory. However, CLP mice treated with TG6–10-1 performed as well as mice in the sham groups.

### EP2 antagonist prevents loss of synaptic protein following sepsis

It is still unknown why administration of EP2 antagonist could prevent memory deficits after CLP. As shown in our previous study, systemic inflammation resulted in reduction of synaptic proteins and EP2 antagonist prevented the reduction of PSD-95 (Jiang Chunxiang, et al., 2020). Here, two synaptic proteins were also measured by western blot. As Fig. 5A, C shows, cortical PSD-95 was reduced to 0.83 ± 0.11 in the CLP mice relative to the appropriately normalized control value at day 7. However, TG6–10-1 reversed the reduction of PSD-95 caused by sepsis. Similar to LPS-treated mice(Jiang Chunxiang, et al., 2020), synaptophysin abundance did not differ significantly among groups (Fig. 5B,D).

### EP2 antagonist does not prevent CCL2 induction in hippocampus

Based on the inflammatory gene profile of hippocampus, CCL2 mRNA was measured with RT-qPCR 7 days after CLP. As expected, CCL2 mRNA was elevated in the hippocampus, but the EP2 antagonist did not inhibit CCL2 mRNA expression (Fig. 6).

## Discussion

Sepsis survivors have a higher risk of developing cognitive impairments and psychological disorders (Iwashyna, et al., 2010;Mazeraud, et al., 2020), yet there is no treatment available. The application of fluid resuscitation and antibiotics help save lives but fail to improve the quality of life after discharge. It is necessary to explore potential therapy to prevent memory deficits following sepsis. Our previous study has shown that EP2 antagonist reverses the reduction of synaptic proteins and prevents memory deficits in the NOR test in a mouse model of sepsis induced by LPS (Jiang Chunxiang, et al., 2020). Here, we further investigated a bacterial sepsis model. We showed that an EP2 antagonist prevented cognitive impairments in the bacterial sepsis model, reinforcing the idea that an EP2 antagonist can improve memory deficits in sepsis models.

The CLP model used here produced a clinically recognizable systemic response, including transient hypothermia, body-weight loss, splenomegaly, altered leukocyte populations, thrombocytopenia, and increased neutrophil-to-lymphocyte and monocyte-to-lymphocyte ratios. NLR is an effective and convenient index that is associated with the prognosis of sepsis (Huang et al., 2020). Importantly, animals received fluid resuscitation and antibiotics to mimic clinical care, yielding a moderate-severity model that facilitated survival to behavioral testing. This design is appropriate for studying consequences among sepsis survivors, although it does not model the full heterogeneity of human sepsis. In particular, only young adult male C57BL/6N mice were studied, and behavioral performance was assessed at one post-sepsis time point. These limitations should be addressed in future work using both sexes, older animals, comorbidity-relevant models, and longer follow-up.

Inflammatory-gene responses differed substantially between the hippocampus and kidney. Kidney cytokine induction was earlier and larger, consistent with a more immediate or robust peripheral inflammatory response. By contrast, hippocampal TNF-α and CCL2 mRNA expression remained elevated at day 7, indicating persistent inflammatory-gene expression in the brain after the acute systemic response had begun to resolve. TG6–10-1 did not significantly alter survival, body weight, Irwin score, splenomegaly, locomotor activity, or hippocampal CCL2 mRNA expression. The cognitive benefit is therefore unlikely to result simply from a broad attenuation of sickness severity or from suppression of CCL2 transcription at day 7. Unlike the status epilepticus model (Jiang J., et al., 2019), the EP2 antagonist failed to inhibit the expression of CCL2 mRNA expression in hippocampus following CLP. Therefore, EP2 receptors may play different roles in modulating the inflammatory response in different neurological disorders.

Cortical PSD-95 abundance was reduced after CLP and preserved in TG6–10-1-treated mice. PSD-95 is a key postsynaptic scaffold that supports glutamatergic synapse organization and plasticity, making it a biologically plausible correlate of impaired recognition memory. However, the present data are correlative and do not establish that PSD-95 loss mediates the cognitive deficit or that its preservation is required for the effect of EP2 antagonism. EP2 signaling can regulate cAMP-dependent cellular processes and has been implicated in synaptic transmission and plasticity (Kim and Sheng, 2004;Wu et al., 2017), raising the possibility that EP2 activation affects PSD-95 trafficking, turnover, or synaptic localization. This hypothesis will require direct testing.

In summary, post-CLP EP2 receptor antagonism prevented impaired novel object recognition and preserved cortical PSD-95 abundance in sepsis-surviving mice without detectably altering several systemic measures of illness or hippocampal CCL2 expression at day 7. These findings nominate EP2 signaling as a plausible therapeutic target for post-sepsis cognitive dysfunction and provide a rationale for defining the relevant neural cell types, signaling events, and therapeutic window.

## Figures and Tables

**Figure 1:**
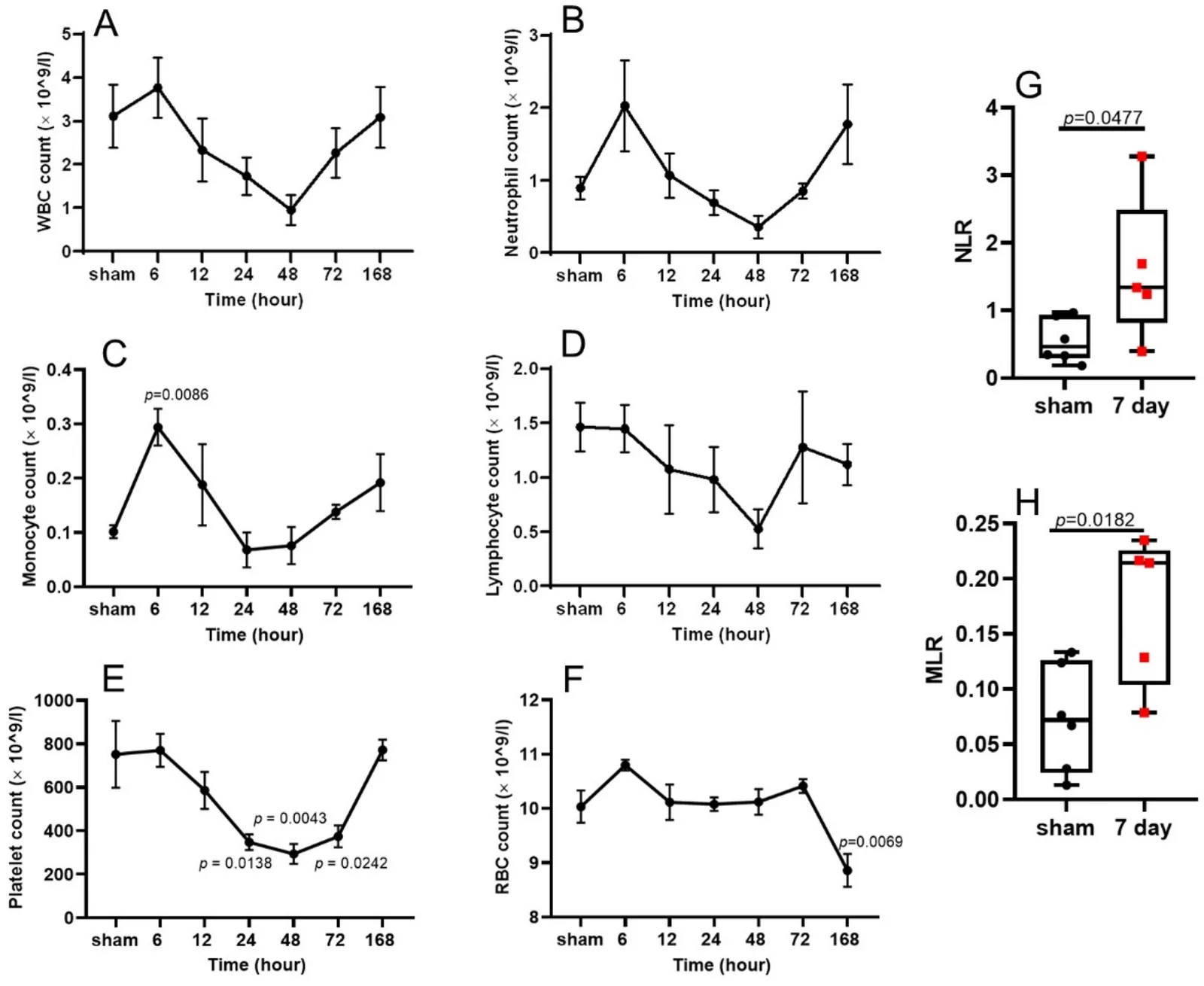
(A-F) Complete blood cell counts were evaluated at the denoted times after sham or CLP surgery, (n = 5–6 mice per time point, compared with sham group, one-way ANOVA and Dunnett’s test). Data are shown as mean ± SEM. (G-H) NLR and MLR at day 7 following CLP procedure were calculated. (n = 5–6 mice per time point, compared with sham group, unpaired t-test). Box‑and‑whisker plots (GraphPad Prism, Min‑to‑Max mode): boxes represent 25th‑75th quartiles, central horizontal line indicates the median; whiskers extend from minimum to maximum observed values.

**Figure 2:**
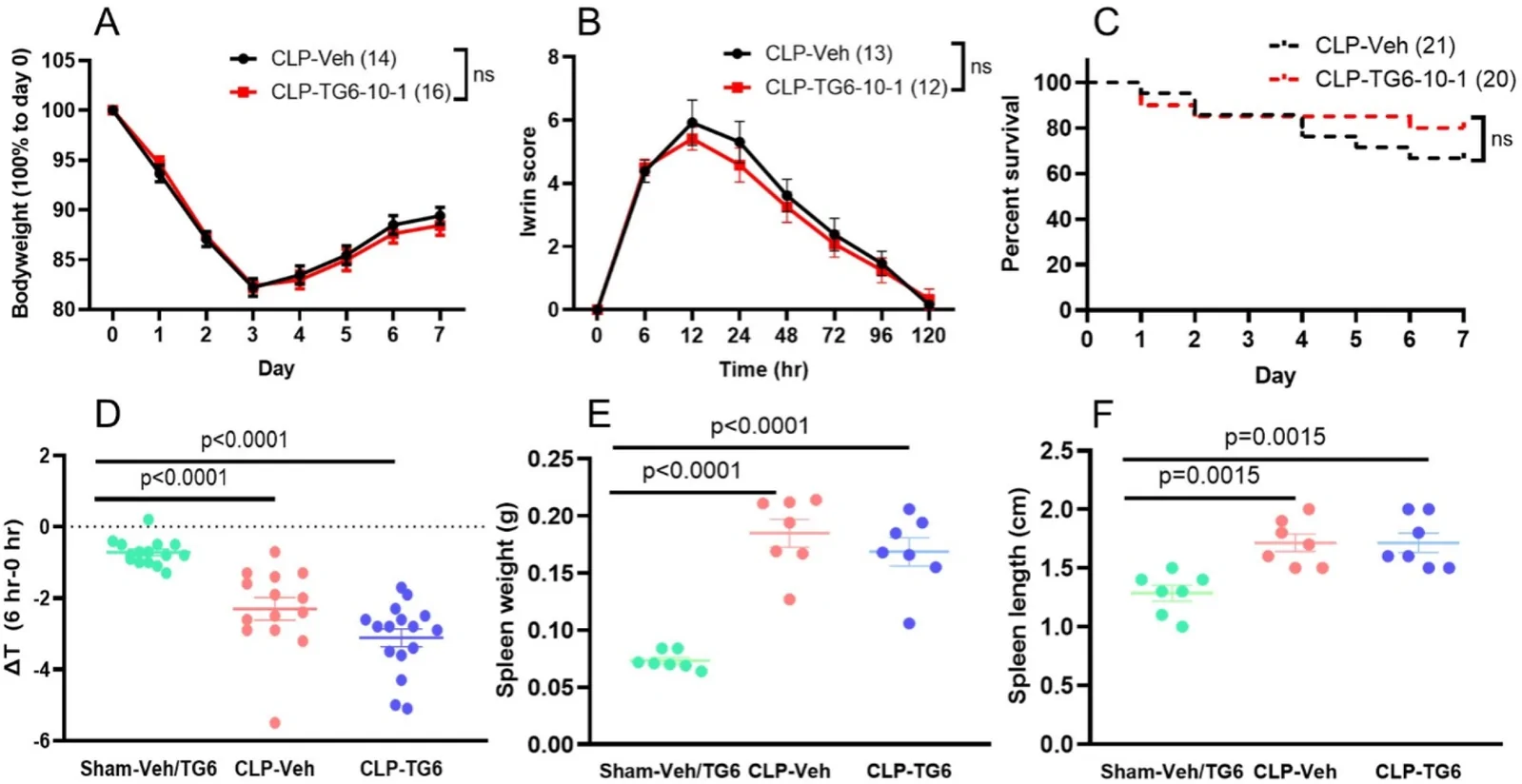
Effect of EP2 inhibition on whole body response to bacterial sepsis. (A) EP2 antagonist did not influence the body weight of septic mice (n = 14 in CLP-Vehicle group, n = 16 in CLP-TG6–10-1 group, ns = *p* ≥ 0.05, two-way ANOVA test). (B) EP2 antagonist did not influence the Irwin score of septic mice (n = 13 in CLP-Vehicle group, n = 12 in CLP-TG6–10-1 group, ns = *p* ≥ 0.05, two-way ANOVA test). (C) EP2 antagonist caused no influence on the survival rate of septic mice (n = 21 in CLP-Vehicle group, n = 20 in CLP-TG6–10-1 group, ns = *p* ≥ 0.05, log-rank (Mantel–Cox test). (D) The temperature at 6 hr following sepsis, (n = 14–16, one-way ANOVA and Dunnett’s test). (E-F) The size and weight of spleen at day 7 following sepsis, (n = 7, one-way ANOVA and Dunnett’s test).

**Figure 3:**
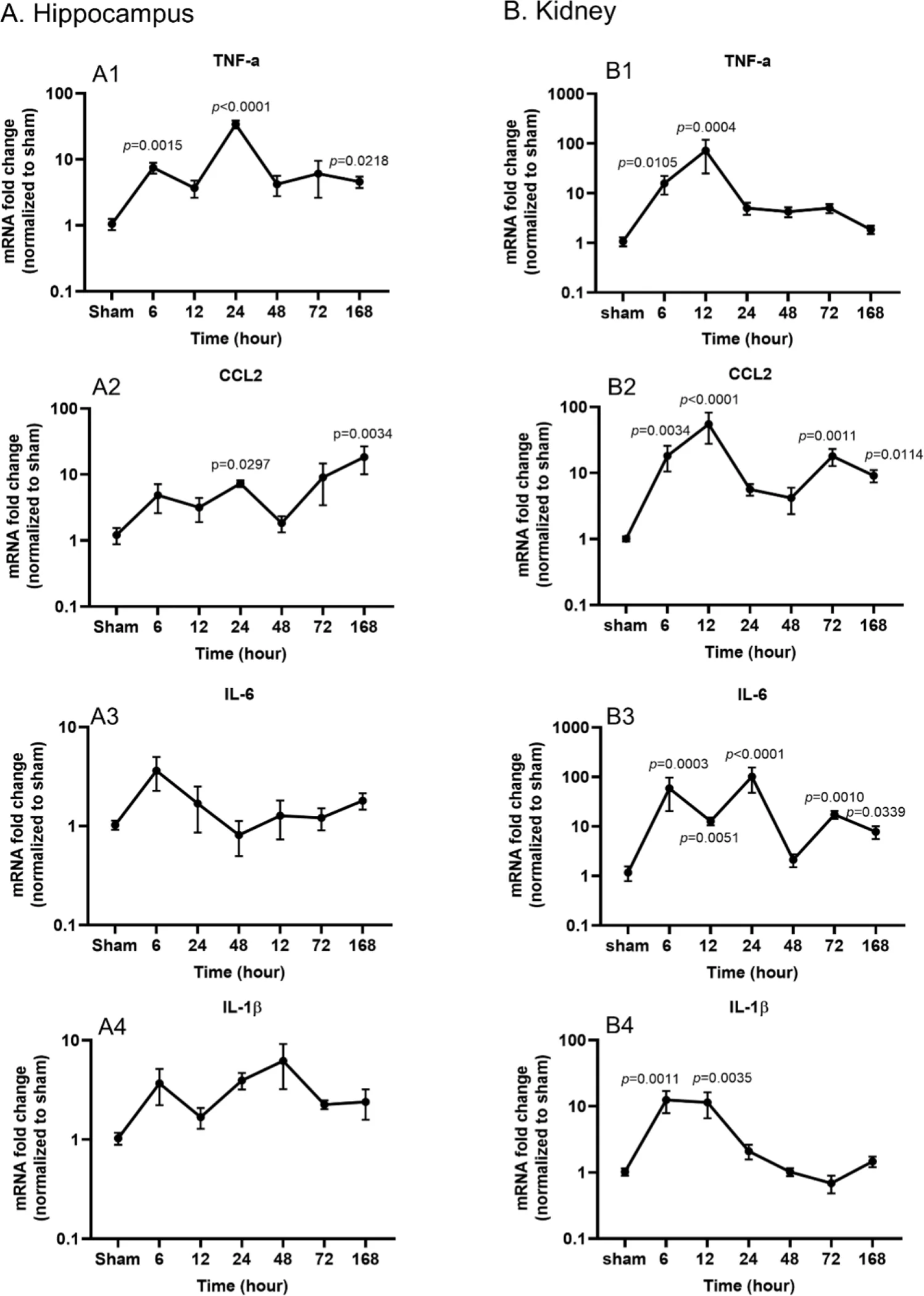
Hippocampus and kidney inflammatory gene expression at the denoted times after sham or CLP surgery, (n = 5–6 mice per time point, compared with sham group, one-way ANOVA and Dunnett’s test). Data are shown as mean ± SEM.

**Figure 4:**
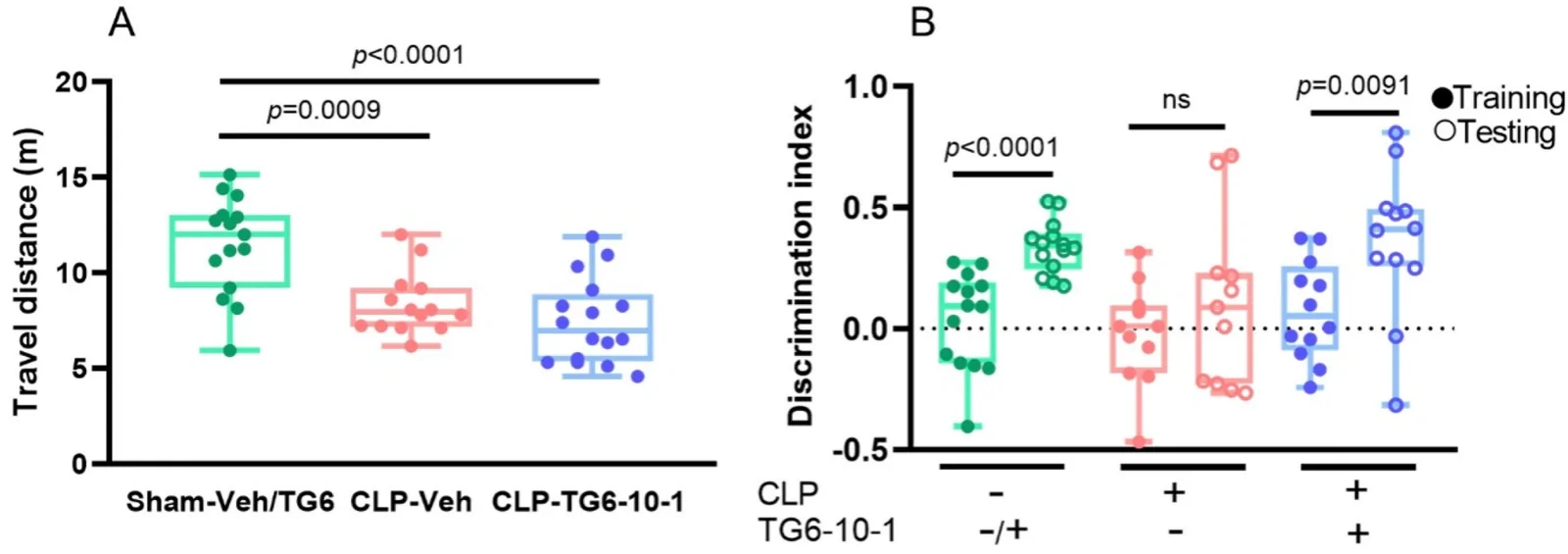
(A) Travel distance on day 7 following sepsis. One‑way ANOVA followed by Dunnett’s multiple comparisons test, exact p‑values are reported in the panel. (B) EP2 antagonist exerts beneficial effects in preventing memory deficits after CLP surgery. n = 11‑14 mice; paired t‑test, within‑animal comparisons for each plotted contrast. Box‑and‑whisker plots (GraphPad Prism, Min‑to‑Max mode): boxes represent 25th‑75th quartiles, central horizontal line indicates the median; whiskers extend from minimum to maximum observed values. Individual animal data points are overlaid. Solid circles, Training; open circles, Testing.

**Figure 5:**
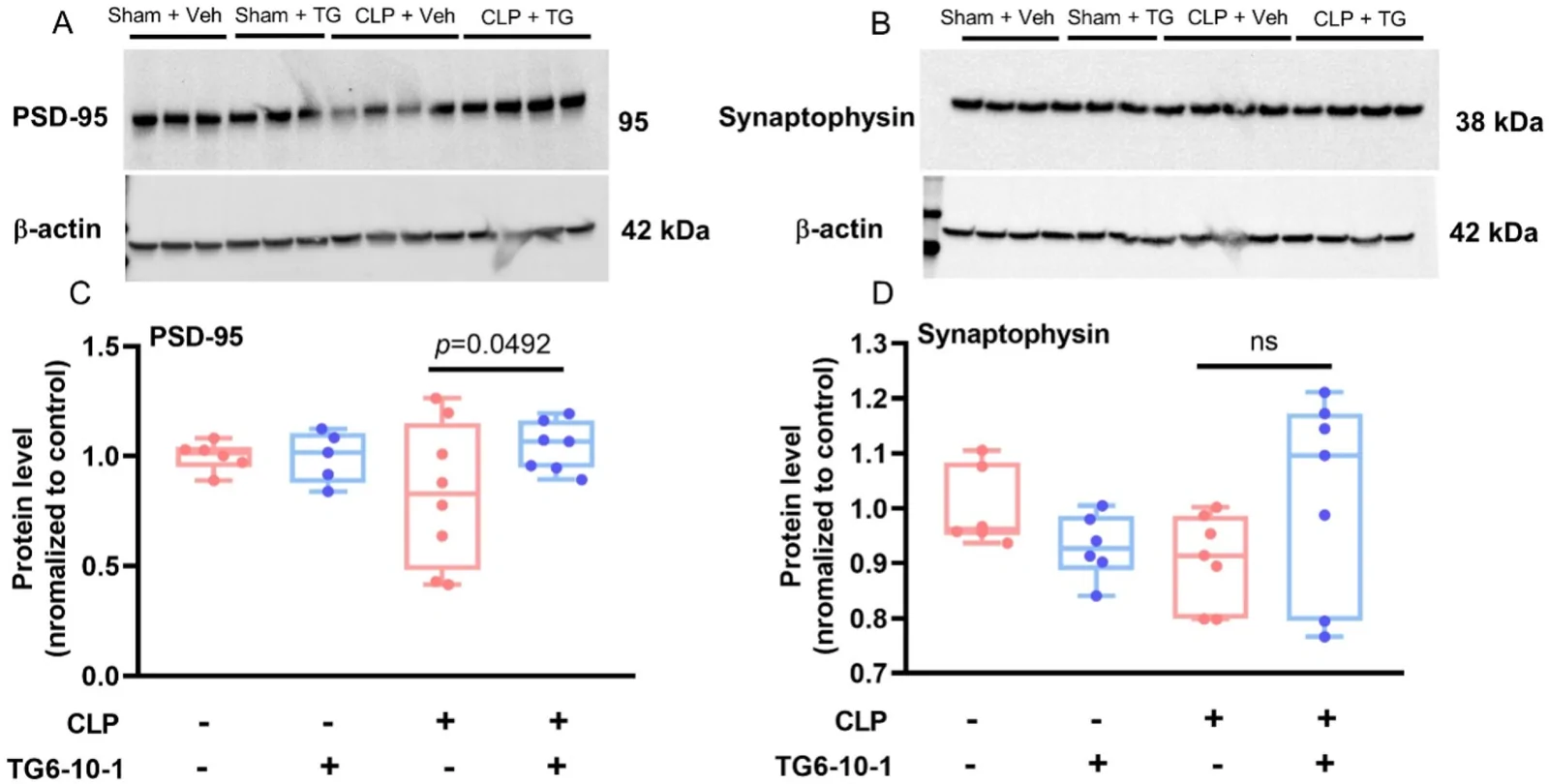
EP2 antagonist reversed the reduction of synaptic protein following sepsis. (A-B) Synaptic protein levels in cortex were measured by western blot. (C) Sepsis reduced PSD-95 expression in cortex, whereas TG6–10-1 reversed this effect, (n = 6–8 mice per group, one-way ANOVA and Bonferroni). (D) Sepsis has no effect on the expression of synaptophysin, (n = 6–8 mice per group, ns = p > 0.05, one-way ANOVA and Bonferroni). Box‑and‑whisker plots (GraphPad Prism, Min‑to‑Max mode): boxes represent 25th‑75th quartiles, central horizontal line indicates the median; whiskers extend from minimum to maximum observed values.

**Figure 6:**
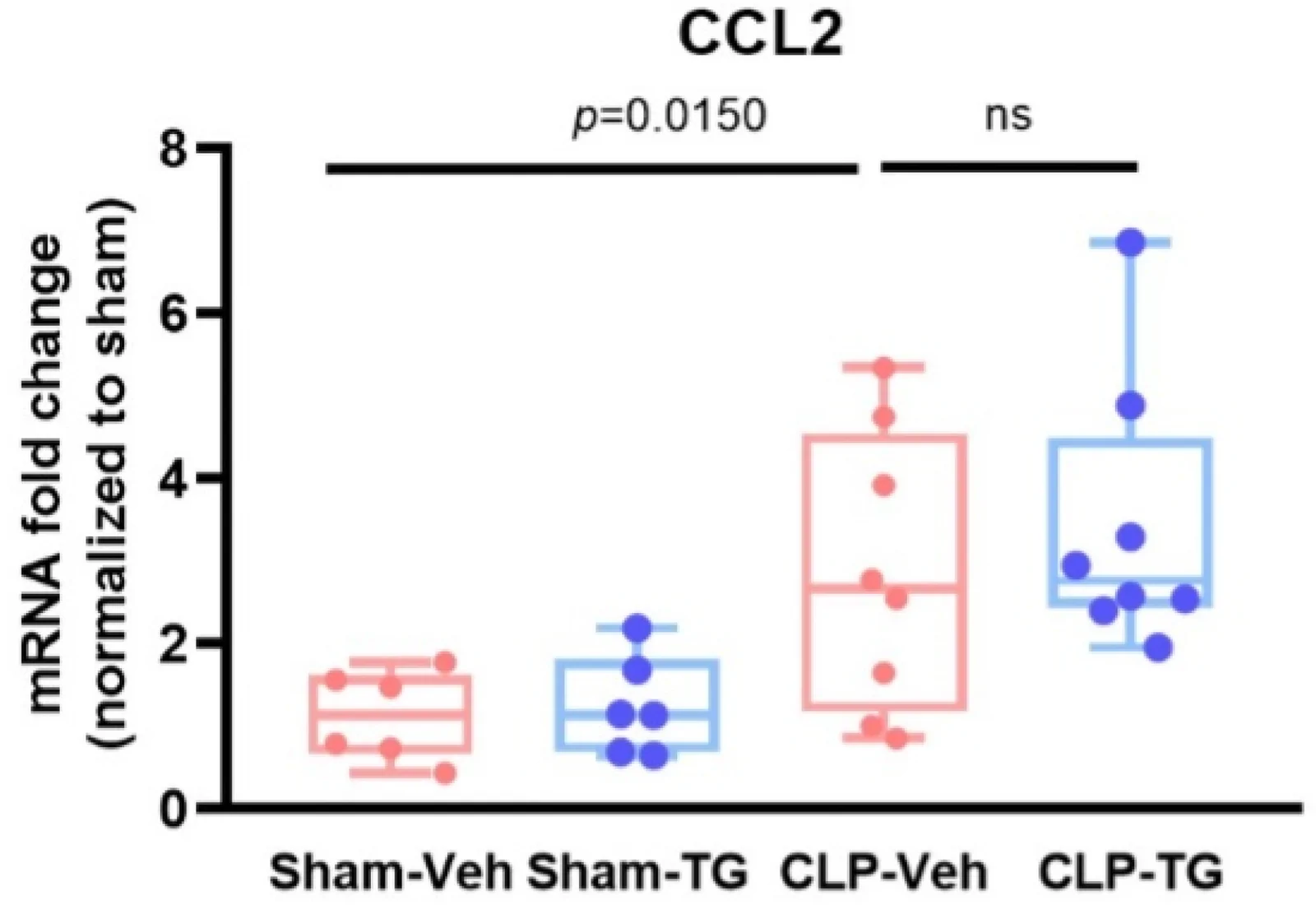
EP2 antagonist did not inhibit expression of CCL2 mRNA in hippocampus after CLP, (n = 6–8 mice per group, one-way ANOVA and Bonferroni). Box‑and‑whisker plots (GraphPad Prism, Min‑to‑Max mode): boxes represent 25th‑75th quartiles, central horizontal line indicates the median; whiskers extend from minimum to maximum observed values.

**Table 1:** Sequence of primers

Genes	Forward Primer (5′-3′)	Reverse primer (5′-3′)
GAPDH	TGTCCGTCGTGGATCTGAC	CCTGCTTCACCACCTTCTTG
β-actin	AAGGCCAACCGTGAAAAGAT	GTGGTACGACCAGAGGCATAC
HPRT1	GGAGCGGTAGCACCTCCT	CTGGTTCATCATCGCTAATCAC
COX-2	CTCCACCGCCACCACTAC	TGGATTGGAACAGCAAGGAT
IL-1β	TGAGCACCTTCTTTTCCTTCA	TTGTCTAATGGGAACGTCACAC
IL-6	TCTAATTCATATCTTCAACCAAGAGG	TGGTCCTTAGCCACTCCTTC
TNF	TCTTCTGTCTACTGAACTTCGG	AAGATGATCTGAGTGTGAGGG
CCL2	CATCCACGTGTTGGCTCA	GCTGCTGGTGATCCTCTTGTA
Iba-1	GGATTTGCAGGGAGGAAAAG	TGGGATCATCGAGGAATTG
CD206	CAAGGAAGGTTGGCATTTGT	CCTTTCAGTCCTTTGCAAGC
Arg1	TCACCTGAGCTTTGATGTCG	CTGAAAGGAGCCCTGTCTTG
